# Wives with long and high-quality hair have more frequent sex

**DOI:** 10.3389/fpsyg.2023.1294660

**Published:** 2024-02-05

**Authors:** Jeong Eun Cheon, Jeongwoo John Kim, Young-Hoon Kim

**Affiliations:** Social and Cultural Psychology Lab, Department of Psychology, Yonsei University, Seoul, Republic of Korea

**Keywords:** hair, hair quality, long hair, sex, sexual intercourse, marriage

## Abstract

The image of an ideal woman often involves her having long, silky hair. However, the dearth of psychological research on hair limits the understanding of how women’s hair functions in romantic relationships. While some scholars have found that the appearance of women’s hair signals reproductive potential to men, whether women’s hair may affect their sexual lives remains unknown. To investigate the function of hair in romantic relationships, we tested whether women’s hair quality and length are associated with sexual frequency in marital dyads. We conducted a paid online survey involving 204 heterosexual marital dyads. The results indicated that women with long and high-quality hair experienced more frequent sexual intercourse with their spouse, as it heightened their husband’s perception of their attractiveness and, consequently, intensified their husband’s sexual desire toward them. Neither men’s hair length nor quality was associated with sexual frequency. Implications of the study are further discussed.

## 1 Introduction

“My thinking was that a girl just is not a girl without her hair.”

(Hemingway, 1979).

For centuries, women have often been depicted as having long, silky hair. Take, for instance, Rapunzel who had a “splendid long hair, as fine as spun gold” (Grimm et al., 1997). Further, in Botticelli’s famous painting *The Birth of Venus*, the goddess of love and beauty Venus is depicted as having beautiful long tresses fluttering in the wind. Such depictions of women with long, silky hair create an image of womanliness, which may be alluring to the perceiver owing to the intricate sense of femininity displayed. In a sense, hair becomes a “symbolic expression of femininity” (Dattagupta, 2018).

Despite the prevailing theme of women’s hair throughout centuries and cultures, surprisingly little psychological research has been conducted to unravel the appealing nature of women’s hair. Following Darwin (1871), who asserted that women’s hair is a factor in sexual selection, a few attempts have been made to associate women’s hair with reproductive potential or attractiveness (Hinsz et al., 2001; Bereczkei and Mesko, 2006), shedding light on the potential role of women’s hair in mating contexts. However, the potential psychological role of hair in romantic relationships remains unclear owing to a lack of thorough investigation on the function of hair.

Whether women’s hair truly signals reproductive potential may become more evident if it touches on the men’s sexual desire toward women with longer and higher-quality hair. This may be supported if further evidence is found of it being associated with frequency of sexual intercourse, either directly or indirectly via influencing men’s sexual desire. If hair is a factor in sexual selection that indicates reproductive potential, hair should elicit positive evaluations and a certain form of sexual response from the (potential) romantic partner. However, with no studies investigating the link between women’s hair and sexual frequency or desire, whether women’s hair may affect their sex lives remains unknown.

To answer the question surrounding the function of women’s hair, this study aims to investigate the effect of hair on couple’s sexual lives. We specifically focus on women’s hair quality and length and their effects on sexual frequency. Greater quality and length of women’s hair may be associated with more frequent sexual intercourse in romantic dyads for three reasons. First, previous studies have shown that lengthy and good quality hair in women is positively evaluated or valued by men (Jackson and McGill, 1996; Grammer et al., 2002; Mesko and Bereczkei, 2004; Sugiyama, 2005; Bereczkei and Mesko, 2006). What is positively evaluated or considered attractive to the opposite sex is more likely to arouse sexual desire in them and suggests an individual’s reproductive potential. Just as optimally pitched voices (Cussigh et al., 2020) and the color red, which evokes an image associated with an elevated level of estrogen and sexual arousal (Elliot and Niesta, 2008), are linked to greater male sexual desire, traits that elicit positive evaluations in men are likely to stimulate men’s sexual desire and imply reproductive potential in women. Hence, it logically follows that a characteristic in women that brings about positive evaluation in men is likely to be associated with greater sexual desire in men. Greater sexual desire in men, in turn, should be associated with a greater amount of sexual intercourse among romantic dyads. This is because sexual desire functions to instigate sexual union or, more distally, reproduction (Fisher, 1998) in romantic dyads.

Second, following the good genes hypothesis (Kokko et al., 2002; Gangestad et al., 2007), people are more likely to desire mates who display indicators of good genetic quality that help increase the fitness of the offspring(s). Women with good genetic quality or reproductive potential signal this quality via a more overt or significant display of certain traits. For instance, large breasts have been shown to communicate higher reproductive potential (Jasieńska et al., 2004; Kościński et al., 2020). This denotes that certain characteristics help attract the opposite sex. Similarly, hair can be one of the indicators in which good qualities are communicated as hair quality has been associated with a perception of good health and youthfulness (Hinsz et al., 2001). Further, it may act as a costly signal that enhances women’s attractiveness as it communicates that they can afford having long hair (Mesko and Bereczkei, 2004). In this regard, hair communicates phenotypic and genetic quality (Mesko and Bereczkei, 2004) that motivates men to reach a reproductive goal. Thus, women’s hair is likely to play a role in motivating men’s sexual desire and should ultimately lead to romantic dyads engaging in more frequent sexual intercourse. That is, the positive evaluations and perceptions of reproductive potential associated with women’s hair quality and length can contribute to increased sexual attraction and, consequently, more active sexual behaviors in couples.

Third, according to according to the Bem’s gender schema theory Bem (1981), traits that align with sex role stereotypes and sexual norms are often rewarded. This principle extends to sexual scripts as well, as those conveying traditional femininity have been found to elicit heightened sexual arousal (Birnbaum et al., 2014). Long hair, historically prevalent among women across cultures like ancient Egypt and Rome (Robins, 1999; Bartman, 2001), is emblematic of this tradition. As such, long and healthy hair has likely become an integral aspect of sexual scripts, serving as a symbol of femininity and attractiveness. The association between long hair and the female prototype (Baktay-Korsós, 2000) reinforces this notion. In a societal context where long and high-quality hair is culturally anticipated for women, men are more inclined to positively evaluate women who possess such hair attributes and subsequently experience heightened sexual desire toward them. Consequently, such favorable evaluations and intensified desire are anticipated to culminate in an elevated frequency of sexual intercourse among romantic couples.

Taken together, we propose that the presence of high-quality, long hair in women should be associated with men’s positive appraisal of women’s attractiveness which, in turn, should lead men to feel greater sexual desire toward women. If a person’s sex drive, having the reproduction-related function which encourages partners to engage in sexual union (Gonzaga et al., 2006), is increased, dyads should have more frequent sexual intercourse. Conversely, men’s hair quality and length are expected to have less impact on dyads’ sexual lives because messages, such as youthfulness, conveyed by hair quality and length may be relatively less valued in men than in women (Hassebrauck, 1998). Additionally, long hair in men may have been perceived as burdensome in hunter-gatherer societies, leading the quality and length of men’s hair to become less emphasized in modern mating contexts.

### 1.1 Overview of the present study

The possible psychological function of hair has never been investigated in committed, romantic relationships. Hence, whether the influence of hair may extend to romantic dyads’ sexual functioning remains to be investigated. This study sought to investigate the function of hair quality and length on sexual frequency in marital dyads. Specifically, women’s hair quality and length were expected to exert a positive effect on sexual frequency by increasing men’s perception of women’s attractiveness and, hence, men’s sexual desire toward them. Men’s hair quality and length were expected to exert a weaker effect on the couple’s sexual frequency. We recruited marital dyads from South Korea to test the hypothesis.

## 2 Method

### 2.1 Participants and procedures

We recruited 204 marital dyads from South Korea (ages ranging from 25 to 51, Wife: *M*_age_ = 33.39, *SD*_age_ = 3.69, Husband: *M*_age_ = 35.77, *SD*_age_ = 4.79) via an online panel service, PanelNow, operated by dataSpring Korea. All dyads were heterosexual couples. They had an average number of 0.9 children, ranging from 0 to 4. The survey was part of a larger study on marital dyads, where variables such as self-personality and perceived spouse personality, which were unrelated to the present research, were examined. For this study, participants answered questions on hair quality, hair length, attractiveness, sexual desire, sexual frequency, perceived partner commitment, relationship satisfaction, and sexual satisfaction. Upon completion of the survey, the dyads were remunerated with approximately 5 USD.

### 2.2 Measures

#### 2.2.1 Hair length

We employed distinct scales to assess hair length in husbands and wives. For husbands, given the lack of existing tools for quantifying men’s hair length, we devised a new single-item scale inspired by a series of representative images depicting various male hair lengths. The scale employed a five-point Likert-type format, where options were as follows: (1) short buzz cut (or shorter), (2) above the eyebrow length, (3) between the eyebrow and the ear length, (4) between the ear and the chin length, and (5) below the chin length. Each category was complemented with a corresponding image to ensure clarity. Similarly, for wives, we presented a five-point Likert-type scale, with each category being supplemented with a relevant image. The categories included: (1) above the ear length (or shorter), (2) between the ear and the chin length, (3) between the chin and the collarbone length, (4) between the collarbone and the chest length, and (5) below the chest length. We referred to the study by Hinsz et al. (2001) for developing the hair length scale for wives. Both husbands and wives were instructed to choose the picture/description that most closely matched their current hair length.

#### 2.2.2 Hair quality

We assessed hair quality using a single-item measure — “Please rate the quality of your hair” — on a seven-point Likert-type scale, ranging from 1 (very bad) to 7 (very good). This decision was driven by several considerations. Primarily, hair quality, while multifaceted in nature, is often perceived by individuals as a singular, holistic attribute rather than a composite of distinct characteristics. Additionally, single-item measures have been validated in the literature as effective tools for capturing global assessments of subjective constructs, particularly when the construct is unambiguous and clearly understood by respondents (Sackett and Larson Jr., 1990; Wanous et al., 1997). Therefore, this single-item measure was employed, allowing participants to directly rate their hair quality.

#### 2.2.3 Physical attractiveness

We asked participants to rate their spouse’s physical attractiveness using a seven-point Likert-type scale (1 = very low, 7 = very high). This rating was adapted from the mate value scale employed by Graham-Kevan and Archer (2009), which aims to measure individuals’ perception of their partner’s value in six domains, namely physical attractiveness, personality, popularity, education, intelligence, and career prospects. For the purpose of our research, we solely focused on the variable of physical attractiveness. As our primary interest was to examine men’s ratings of their spouse’s attractiveness, we considered the other five items—personality, popularity, education, intelligence, and career prospects—irrelevant to our study. Therefore, we specifically used the physical attractiveness rating of the participants’ spouses to assess the perception of their partner’s physical appeal.

#### 2.2.4 Sexual desire toward spouse

To measure participants’ specific sexual desire for their spouse, we modified the scale originally proposed by Tidwell and Eastwick (2013). The original scale includes two items that measure the general sex drive of participants (i.e., “I have a strong sex drive” and “I experience sexual desire extremely frequently.”). We modified these items to measure sex drive toward spouses (i.e., “I have a strong sex drive toward my partner” and “I experience sexual desire extremely frequently toward my partner.”). Participants were asked to answer each item using a seven-point Likert-type scale (1 = strongly disagree, 7 = strongly agree). The Spearman–Brown reliability coefficient was 0.86 for husbands and 0.92 for wives.

#### 2.2.5 Sexual frequency

Following Park et al. (2023), we developed a single-item measure to assess frequency of monthly sexual intercourse with the spouse. Participants were asked the following: “On average, how many times per month do you have sexual intercourse with your partner?” Participants were asked to provide a numeric estimate of their monthly sexual frequency.

#### 2.2.6 Control variables

In this study, we controlled for several variables to account for potential confounding factors and better isolate the effects of hair quality and length on sexual frequency in romantic dyads. First, we controlled for the age of wife and husband, as previous research has suggested a tendency for younger women to have longer hair (Hinsz et al., 2001). Additionally, age has been found to be significantly related to sexual frequency (Karraker et al., 2011). By controlling for age, we aim to eliminate any potential influence of age-related factors on the relationship between hair attributes and sexual frequency.

Second, marital and sexual satisfaction were included as control variables, given their close association with the frequency of sexual intercourse (McNulty et al., 2016). Marital satisfaction was measured using six items from the Quality Marriage Index by Norton (1983). Sexual satisfaction was measured using the Global Measure of Sexual Satisfaction (Lawrance and Byers, 1998), consisting of five items. Both scales utilized a seven-point Likert-type format and demonstrated excellent reliability (Cronbach’s *α* = 0.95 and 0.96 for husbands’ and wives’ marital satisfaction, respectively; 0.94 and 0.93 for the sexual satisfaction levels of husbands and wives, respectively). By considering these satisfaction levels, our aim was to ensure that the observed effects of hair quality and length on sexual frequency were not confounded by overall relationship satisfaction.

Furthermore, we controlled for the perceived level of commitment reported by both the wife and husband toward each other. This control is important because long, high-quality hair may not have a positive effect on the sexual lives of romantic dyads if these characteristics are perceived by the spouse as attracting other potential mates. We measured perceived commitment level using an adapted version of the seven-item scale from Rusbult et al. (1998). The reliability scores of the measure were 0.90 and 0.92 for husbands and wives, respectively. By incorporating perceived commitment levels, our intention was to refine our understanding of the interplay between hair attributes and sexual frequency.

Additionally, the duration of marriage and the number of children were included as dyadic level variables, as they can also influence sexual frequency (Call et al., 1995). By controlling for these factors, we aimed to better isolate the specific impact of hair quality and length on sexual frequency among romantic dyads.

## 3 Analytic strategy

In this study, we used an adapted version of the actor–partner interdependence model (APIM) approach within the structural equation modeling framework, following the methodology proposed by Kashy and Kenny (2000). The dyadic dataset was analyzed using path analysis in Mplus version 8.7. The APIM is a statistical framework designed to investigate the interdependence between individuals within dyadic relationships, such as romantic couples. By adopting this approach, we could account for the mutual influence that partners have on each other’s outcomes while simultaneously estimating actor effects (i.e., the impact of one’s own characteristics on their own outcome) and partner effects (i.e., the impact of one partner’s characteristics on the other partner’s outcome).

Specifically, to examine the interconnectedness of variables within the dyad, we calculated residual covariances between wives’ and husbands’ mediator variables. Additionally, covariances between all endogenous variables were computed. We estimated the saturated model with a perfect fit.

To assess the indirect effect of hair quality and length on sexual frequency, we employed the bias-corrected bootstrapping method, as recommended by MacKinnon et al. (2004) and Lau and Cheung (2012). This method is well-suited to handle non-normal data and provides robust estimates of indirect effects. The bootstrapping procedure involved 5,000 resamples, and the 95% confidence interval (CI) was used to determine the significance of the indirect effect.

Furthermore, we conducted a comparison between husbands and wives to explore potential gender differences in the indirect pathway (Diff). This comparison allows us to investigate whether hair quality and length have differential impacts on sexual frequency for men and women within the romantic dyads. By employing the APIM and conducting thorough statistical analyses, we aimed to gain a comprehensive understanding of the intricate dynamics within romantic relationships and how hair attributes are associated with sexual frequency. Data and analysis codes are accessible through OSF at https://osf.io/fhgyn/. Additionally, the results without covariates are also available at the OSF link.

## 4 Results

### 4.1 Sexual frequencies

The sexual frequencies as reported by husbands and wives were 3.58 and 3.30, respectively. The frequencies were significantly correlated (*r* = 0.83, *p* < 0.001), indicating a strong relationship between the variables. As suggested by Ferguson (2016), this correlation value well surpassed the practical significance threshold of 0.2. To enhance the reliability of responses provided by the dyads, we proceeded to analyze the discrepancy between the reports of monthly sexual frequency for wives and husbands (*M* = 0.98, *SD* = 1.59). The discrepancy between wife’s and husband’s report of sexual frequency ranged from 0 to 14, with the majority of dyads (78.4%) yielding a discrepancy score of 0 or 1. We removed the three outliers that exceeded three standard deviations away from the mean (discrepancy scores being 6, 7, and 14, respectively) from the subsequent analysis. However, the inclusion of those three dyads does not change the pattern of the findings. After excluding three dyads, the correlation between the sexual frequencies reported by the husbands and wives was 0.89 (*p* < 0.001). The sexual frequency as reported by husbands and wives did not differ (*t*(201) = −1.49, *p* = 0.147, 95% CI = [−0.351, 0.053]). Hence, following previous studies (Gager and Yabiku, 2010; Park et al., 2023) and to improve the construct’s reliability, we computed average sexual frequency by averaging the husband’s and wife’s reports of sexual frequency. Additionally, we performed a log transformation on the sexual frequency variable by adding 1 to address its skewness and account for a potential threshold effect. Previous studies have suggested that sexual activity may demonstrate a threshold effect, wherein the influence of increasing sexual activity is more prominent at lower levels than at higher levels (Blanchflower and Oswald, 2004). This log transformation method, also used in prior research on sexual frequency (Gager and Yabiku, 2010), allows us to ensure a more appropriate distribution of the data for statistical analyses.

### 4.2 Preliminary analyses

The results of the correlation among all key variables, including the covariates, are shown in Table 1. As shown in the table, there was a significant positive relationship between wife’s hair quality and average sexual frequency (*r* = 0.16, *p* = 0.023). However, wife’s hair length and average monthly sexual frequency were not significantly correlated (*r* = 0.09, *p* = 0.188). A similar pattern was observed for husband-rated wife’s attractiveness. That is, a significant positive relationship was observed between the wife’s hair quality and husband-rated wife’s attractiveness (*r* = 0.20, *p* = 0.004). However, there was no significant correlation between wife’s hair length and husband-rated wife’s attractiveness (*r* = 0.09, *p* = 0.187).

**Table 1:** Correlation of measured variables.

Variable	1	2	3	4	5	6	7	8	9	10	11	12	13	14	15	16	17
1. SF																	
2. WHL	0.09																
3. WHQ	0.16*	−0.09															
4. WA	0.06	0.09	0.20**														
5. WSD	0.49**	−0.02	0.14*	0.08													
6. HHL	0.09	0.10	0.06	0.08	0.09												
7. HHQ	0.05	0.01	0.11	0.14	0.07	0.04											
8. HA	0.08	−0.03	0.11	0.03	0.18**	0.09	0.20**										
9. HSD	0.37**	−0.07	−0.01	0.40**	0.28**	0.09	0.06	−0.40									
10. MARL	−0.16*	−0.14*	−0.07	−0.22**	−0.28**	−0.06	0.06	−0.05	−0.17*								
11. Wage	−0.28**	−0.27**	−0.00	−0.12	−0.26**	−0.21**	−0.02	−0.15*	−0.17*	0.54**							
12. WMsat	0.28**	0.01	0.18**	0.25**	0.45**	0.06	0.17*	0.28**	0.27**	−0.33**	−0.13						
13. WSexSat	0.40**	0.02	0.27**	0.18**	0.52**	0.02	0.18*	0.27**	0.21**	−0.26**	−0.19**	0.50**					
14. WHCOM	0.29**	0.03	0.14*	0.26**	0.38**	0.08	0.08	0.18*	0.37**	−0.28**	−0.19**	0.70**	0.41**				
15. Hage	−0.24**	−0.14*	−0.07	−0.15*	−0.22**	−0.19**	−0.01	−0.10	−0.22**	0.58**	0.71**	−0.21**	−0.20**	−0.24**			
16. HMsat	0.16*	−0.03	0.16*	0.48**	0.28**	0.10	0.19**	0.12	0.39**	−0.26**	−0.09	0.71**	0.33**	0.61**	−0.18*		
17. HSexSat	0.39**	−0.02	0.10	0.43**	0.39**	0.05	0.27**	0.09	0.46**	−0.18**	−0.16*	0.42**	0.38**	0.41**	−0.15*	0.57**	
18. HPCOMM	0.21**	−0.01	0.07	0.30**	0.28**	0.13	0.11	0.15*	0.33**	−0.22**	−0.16*	0.61**	0.30**	0.60**	−0.18*	0.69**	0.50**

SF, Sexual frequency (log transformed); WHL, Wife’s hair length; WHQ, Wife’s hair quality; WA, Wife’s attractiveness (husband rating); WSD, Wife’s sexual desire; HA, Husband’s attractiveness (wife rating); HSD, Husband’s sexual desire; MARL, marital length; Wage, Wife’s age; WMsat, Wife’s marital Satisfaction; WSexSat, Wife’s sexual satisfaction; WHCOM, Wife-reported husband’s commitment; Hage, Husband’s age; HMsat, Husband’s marital satisfaction; HSexSat, Husband’s sexual satisfaction; HWCOM, Husband-reported wife’s commitment.

*p < 0.05. **p < 0.01.

Neither husband’s hair quality (*r* = 0.05, *p* = 0.515) nor length (*r* = 0.09, *p* = 0.187) was significantly related to average sexual frequency. Husband’s hair quality was significantly associated with wife-rated husband’s attractiveness (*r* = 0.20, *p* = 0.004). However, husband’s hair length was nonsignificantly associated with wife-rated husband’s attractiveness (*r* = 0.09, *p* = 0.206).

### 4.3 Main analyses

**Figure 1:**
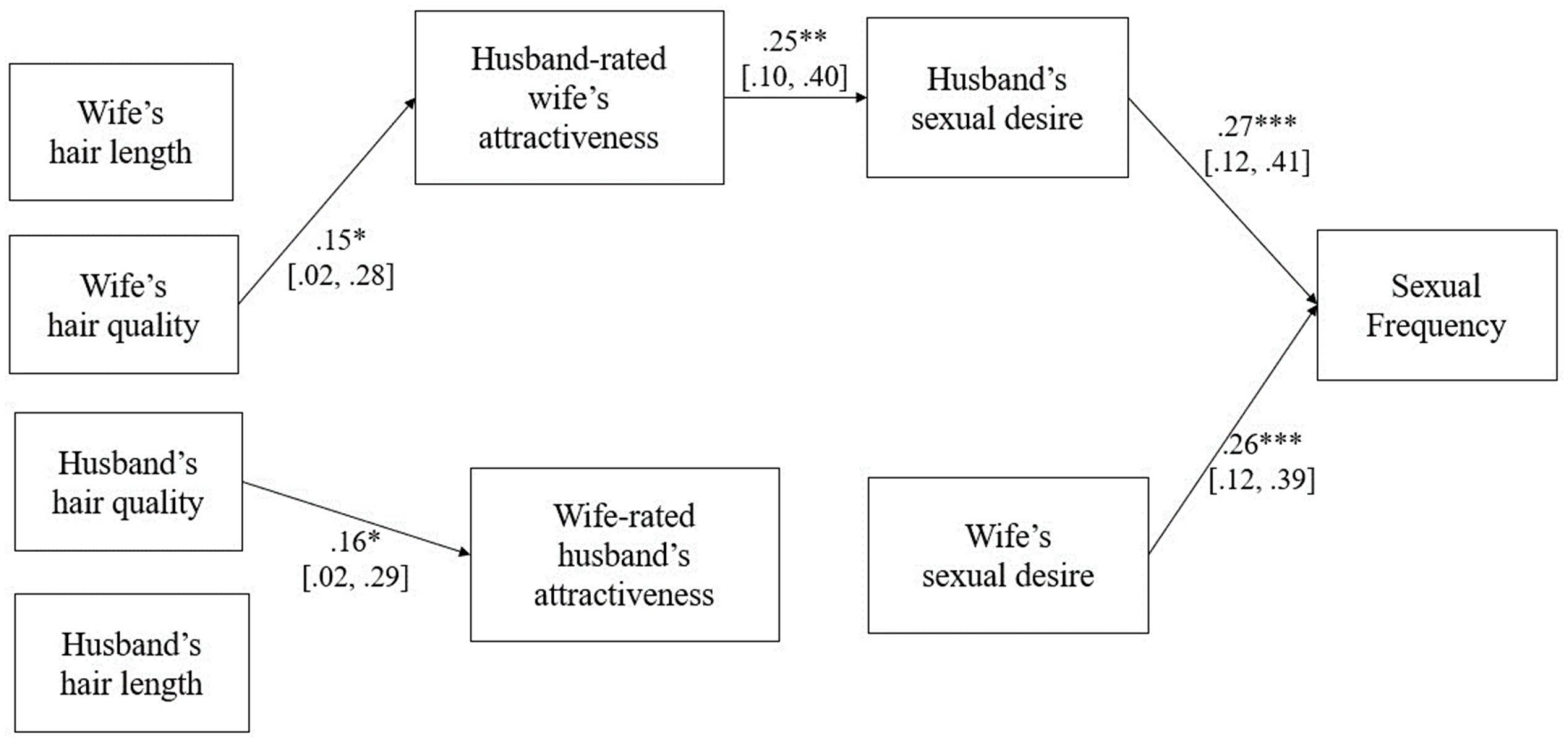
Mediation model tested. Standardized regression coefficients are presented exclusively for significant paths where the CI does not include 0. Estimates for nonsignificant paths, correlations between endogenous variables, and correlation between residual variances are not shown. Control variables are not shown. The upper and lower bounds of 95% bias-corrected bootstrap CI are shown in square brackets. *p < 0.05. **p < 0.01. ***p < 0.001.

This study tested for the adapted version of the double mediation APIM model (Figure 1). An examination of the absolute skewness and kurtosis values indicated that there was no substantial violation of normality for all the variables examined (i.e., skewness < |2|, kurtosis < |7|; Kim, 2013).

First, we examined whether hair quality and length had a direct effect on sexual frequency, controlling for wife’s and husband’s attractiveness and sexual desire. The results of our analysis indicated trends toward a positive association between wife’s hair quality and sexual frequency (*β* = 0.13, *SE* = 0.07, 95% CI = [−0.008, 0.277], *p* = 0.070). Similarly, a comparable trend was observed between wife’s hair length and sexual frequency (*β* = 0.11, *SE* = 0.06, 95% CI = [−0.002, 0.223], *p* = 0.051). However, husband’s hair quality (*β* = −0.05, *SE* = 0.06, 95% CI = [−0.170, 0.070], *p* = 0.430) and hair length (*β* = 0.01, *SE* = 0.06, 95% CI = [−0.111, 0.141], *p* = 0.829) were not significantly associated with sexual frequency.

Next, we examined the indirect effect of hair quality on average sexual frequency via partner’s rating of their spouse’s attractiveness and partner sexual desire. Table 2 shows that the wife’s hair quality–wife’s attractiveness–husband’s sexual desire–sexual frequency link was significant. This indicates that a wife’s hair quality increases their attractiveness and, in turn, increases their husband’s sexual desire toward them. Higher husband’s sexual desire, in turn, leads dyads to have more frequent sexual intercourse. When we tested for the comparable indirect pathway from husband’s hair quality to sexual frequency, the path was nonsignificant (Table 2). Further, we tested whether the strength of the indirect effect significantly differ for wives and husbands. The result revealed that the effect of both wives’ and husbands’ hair on sexual frequency did significantly differ (Diff = 0.01, *SE* = 0.00, 95% CI = [0.000, 0.015]).

**Table 2:** Indirect pathway predicting sexual frequency.

Indirect path	*β*	*SE*	*z*	[CI 95% bias-corrected]
**WHQ→WA→HSD→SF**	**0.01**	**0.01**	**1.64**	**[0.002, 0.027]**
HHQ → HA → WSD → SF	0.00	0.00	0.05	[−0.007, 0.007]
**WHL→WA→HSD→SF**	**0.01**	**0.01**	**1.32**	**[0.000, 0.024]**
HHL → HA → WSD → SF	0.00	0.00	0.03	[−0.003, 0.005]

WHQ, Wife’s hair quality; WA, Wife’s attractiveness (husband rating); HSD, Husband’s sexual desire; SF, Sexual frequency (log transformed); HHQ, Husband’s hair quality; HA, Husband’s attractiveness (wife rating); WSD, Wife’s sexual desire; WHL, Wife’s hair length; HHL, Husband’s hair length. CI 95%, 95% confidence interval [lower-bound, upper-bound]. Significant effects are displayed in bold.

When we examined the indirect effect of hair length on average sexual frequency, the influence of wife hair length on sexual frequency through husband-rated wife’s attractiveness and husband’s sexual desire proved to be statistically significant (Table 2). Although the relationship between wife’s hair length and husband-rated wife’s attractiveness showed marginal significance (*β* = 0.11, *SE* = 0.06, 95% CI = [−0.014, 0.230], *p* = 0.070), the indirect effect remained significant.^1^ On the other hand, the indirect pathway from husband’s hair length to sexual frequency via wife-rated husband’s attractiveness and wife’s sexual desire did not yield significance. The strength of the indirect effect from hair length to sexual frequency significantly differed for wives and husbands (Diff = 0.01, *SE* = 0.00, 95% CI = [0.000, 0.018]). Neither marital length nor commitment level moderated the effects, suggesting that the influence of the wife’s hair quality or length on sexual frequency remained consistent regardless of marital length or commitment level (all *p*s > 0.05).

## 5 Discussion

There is a captivating quality to a woman’s long, healthy hair. Practically, having shiny long hair does not seem to have direct evolutionary benefits. However, women invest considerable amounts of time caring for and grooming their hair. What function could women’s hair have? Building on theories on evolutionary psychology and sexual schema, this study showed that women’s hair is related to sexual signaling systems, increasing pair bonding between romantic dyads.

Specifically, we observed a positive association between women’s hair quality and sexual frequency among marital dyads. This suggests that women’s hair quality may serve as an important cue for sexual attractiveness within romantic relationships. Further examination demonstrated that women’s hair quality influenced men’s ratings of women’s attractiveness, subsequently arousing greater sexual desire in men toward their partners. Greater sexual desire in men was subsequently associated with a higher likelihood for marital dyads to have more frequent sexual intercourse. Likewise, the length of women’s hair displayed a similar trend. Men found women with longer hair to be more attractive, which consequently resulted in heightened sexual desire among men. This heightened sexual desire was again associated with a higher likelihood of more frequent sexual intercourse within the couples.

While both women’s hair quality and length were associated with sexual frequency in marital dyads, hair quality overall showed a correlation with sexual frequency. This suggests that hair quality, rather than length, might more effectively signal a woman’s innate attractiveness or reproductive potential. Notably, hair quality, being less controllable than hair length, is potentially a more innate indicator of a woman’s mate value or genetic quality. Although long hair on women has been linked to greater attractiveness (Mesko and Bereczkei, 2004), shorter hair on women can denote positive traits such as caring and emotional strength (Bereczkei and Mesko, 2006). In contexts where factors like parenting are considered, hair length may not play as significant a role as it does in the pursuit of romantic partners. Moreover, unlike hair length — which can be easily altered through cutting, trimming, or growing — hair quality can indicate more fundamental aspects like good health or the absence of illness (Goldberg and Lenzy, 2010). Due to these distinctions, hair quality may serve as a more reliable indicator of reproductive potential. Consequently, hair quality might have a more pronounced effect on men’s evaluations of women and the frequency of sexual activity within dyads. To further understand these nuances, future research should investigate how various combinations of hair length and quality affect sexual frequency.

By revealing the intricate relationship between hair, attractiveness, and sexual activity, our study contributes to the understanding of physical attractiveness in a broader sense. Although physical attractiveness has been extensively investigated concerning facial neonate features (Jones et al., 1995) or waist-to-hip-ratio (Henss, 2000), hair has not received enough attention from scholars. This study highlights that hair may be vital in assessing women’s attractiveness or reproductive potential. Along these lines, our study implies that the investment women make in their hair care and grooming could be indicative of their importance in attracting and maintaining romantic partners. Understanding the interplay between physical attractiveness, sexual desire, and sexual activity contributes to a more comprehensive understanding of human behavior and relationships.

Moreover, the act of displaying hair quality by a woman may also play a role in increasing a man’s sexual desire for her. Various hairstyles and ways of presenting one’s hair can impact perceptions of attractiveness (e.g., Janif et al., 2015). For example, the act of slicking one’s hair back or untying one’s tied hair may be associated with how people perceive the individual’s attractiveness. Historical associations have linked tied hair to restrained sexuality (Leach, 1958), implying that untying one’s hair may signal a change in sexual self-presentation. These nuances in hair presentation and the subsequent impact on sexual desire warrant further investigation. In fact, this aspect of hair presentation opens up intriguing avenues for future research. More studies are needed to investigate how variations in hair quality, along with changes in its presentation, might influence mating behaviors and sexual activities. Understanding the interplay between hair quality, hairstyles, and their impact on attraction and sexual desire could shed light on cultural practices related to women’s hair, such as the wearing of hijabs.

Our findings imply that women’s characteristics known to attract potential mates further work to influence the romantic lives of marital dyads. Although features associated with sexual selection have been focused mainly in non-established relationships, this study calls for more psychological studies to investigate how men’s and women’s traits that denote higher mate value may influence marital satisfaction of marital dyads. That is, characteristics linked to sexual selection do not seem to merely attract potential mates. Beyond the initial function, it seems to help hold marital partners together. This finding is significant in that it showed how physical features associated with reproductive potential exert a significant effect in sexual lives of marital dyads.

However, it is essential to contextualize that the influence of hair on the romantic dynamics of marital dyads was relatively modest. A correlation of women’s hair with sexual frequency indicated a small effect size. After accounting for factors like age, perceived attractiveness, satisfaction, commitment, and others, its direct association with sexual frequency remained small. The indirect influence of hair length and quality on sexual frequency was very small. Without other control variables entered in the analyses, only the direct effects of women’s hair quality and length proved to be significant. This finding suggests that hair attributes might subtly influence sexual dynamics, operating more on an implicit level to trigger sexual response systems rather than directly shaping conscious sexual decision-making. Further, the small effect size underscores that there are likely other more influential factors affecting sexual dynamics, pointing to a somewhat limited overall effect of hair characteristics. Additionally, given the cross-sectional nature of our study, caution must be exercised in interpreting these findings. The reliance on a single-item scale to measure hair quality and length also suggests a need for more comprehensive measurement methods in future research.

Stepping further, future research should explore how the importance of hair quality or length may vary across different demographics, motivations, or stages of life. Although our study did not find significant interactions between these hair attributes and either marital duration or commitment level, individual preferences might still play a critical role. For example, some individuals may prefer shorter hair on women, with these preferences potentially changing over time or as they move through different life phases. Similarly, the preferences and behaviors of couples in the nascent stages of dating could differ from those in longer, more established relationships. Such potential variations in individual preferences merit further investigation in future studies.

Given that hair has often been a neglected topic, especially in marital dyads, this study is meaningful in that it established the positive relationship between hair quality and sexual frequency. This finding denotes that women’s hair may, indeed, work to convey an evolutionary message to the opposite sex. Further, considering that sexual frequency has declined among American adults (Twenge et al., 2017), an investigation of factors related to greater sexual frequency opens a promising avenue where interventions for having more positive sexual lives can be promoted.

## 6 Conclusion

This study investigated whether hair is associated with attractiveness and sexual lives in romantic dyads. The results revealed a positive effect of women’s hair quality and length on sexual frequency in romantic dyads. Wives’ long and high-quality hair was associated with frequent sexual intercourse via increasing their attractiveness and, subsequently, their husbands’ sexual desire toward them. This indicates that wives’ long and high-quality hair may arouse positive evaluation as well as sexual desire in husbands, thereby promoting pair bonding in couples.

## Data availability statement

The datasets presented in this study can be found in online repositories. The names of the repository/repositories and accession number(s) can be found at: https://osf.io/fhgyn/.

## Ethics statement

The study was conducted in accordance with the Declaration of Helsinki and approved by the Institutional Review Board (or Ethics Committee) of Yonsei University (IRB no. 7001988-202207-HR-1605-03). The studies were conducted in accordance with the local legislation and institutional requirements. The ethics committee/institutional review board waived the requirement of written informed consent for participation from the participants or the participants’ legal guardians/next of kin because as this study involved an online survey with minimal anticipated harm to participants, we obtained informed consent through an online consent form.

## Author contributions

JC: Conceptualization, Data curation, Formal analysis, Investigation, Methodology, Writing – original draft. JK: Investigation, Validation, Writing – review & editing. Y-HK: Project administration, Supervision, Validation, Writing – review & editing.

## Footnotes

^1^It is important to note that an indirect effect can attain significance even if individual indirect paths lack significance (Zhao et al., 2010; Jollineau and Bowen, 2022).
